# Early Relational Health and its Impact on the Developing Brain: A Scoping Review

**DOI:** 10.1007/s10567-025-00545-3

**Published:** 2025-09-17

**Authors:** Lu Zhang, Daniel Liontos, Craig A. Olsson, Tracy Evans-Whipp, Jennifer. E. McIntosh, Felicity Painter, Jacquelyn Harverson, Sarah Whittle, The Australian Early Relational Health Network

**Affiliations:** 1 Centre for Youth Mental Health, University of Melbourne, 35 Poplar Road, Parkville, VIC 3052 Australia; 2 Orygen, Parkville, VIC Australia; 3 Faculty of Health, School of Psychology, SEED Lifespan Strategic Research Centre, Deakin University, Victoria, Australia; 4 Centre for Adolescent, Murdoch Children’s Research Institute, Melbourne Royal Children’s Hospital, Victoria, Australia; 5 Department of Paediatrics, Melbourne Royal Children’s Hospital, The University of Melbourne, Victoria, Australia; 6 The Bouverie Centre, La Trobe University, Bundoora, VIC Australia

**Keywords:** Relational health, Mother-infant, Parent-infant, Neurobiology, Brain development

## Abstract

The formation of secure parent–child bonds in early life, hereto referred to as early relational health, plays a central role in healthy development. However, the evidence on how early relational health ‘gets under the skin’ to impact the developing brain remains unclear. Here, we provide a scoping review of the extant literature and synthesize evidence on the link between relational health from conception to age three and subsequent brain structure, function, and connectivity. Literature searches in MEDLINE, PsycINFO, and Embase databases yielded 7156 studies. Screening of studies was conducted on the Living Knowledge System (an AI-assisted screening tool), which resulted in 79 studies being included in the review. Results were synthesized based on the type of relational health examined, type of imaging modality (e.g., electroencephalogram, structural, and functional magnetic resonance imaging), and developmental stage. We identified studies of the relationship between early relational health and brain structure, function, and connectivity that spanned from childhood to young adulthood, with no research beyond this age period. There was evidence for early relational health to be associated with patterns of brain activation that may reflect the experience of more positive emotions and reduced risk for psychopathology. However, few studies examined longitudinal changes in brain structure, function, and connectivity. Even fewer have examined relational health beyond the mother–infant bond. Future research is needed to improve understanding of the impact of relational health on brain development and to ascertain whether such impacts reflect a mechanism linking relational health with health and wellbeing across the lifespan.

**Supplementary Information:**

The online version contains supplementary material available at 10.1007/s10567-025-00545-3.

## Introduction

Early relational health, as defined by children’s ability to form safe, stable, and nurturing relationships, is central to a healthy start to life (Frosch et al., 2021). A relational focus, above existing constructs of solely attachment or family functioning, offers a more comprehensive and child-centered conceptualization (Ainsworth, 1978; Epstein et al., 1983; Frosch et al., 2021). While the focal point of attachment theory is the child’s emotional security and attachment behaviors with their primary caregiver, relational health captures a broader, ongoing construct (Ainsworth, 1978). It can include relationships beyond the typical dyad setting (e.g., within larger family structures, community) and highlights the child’s continuing capacity to engage in and sustain their relationships (Frosch et al., 2021). It also differs from family functioning and parental styles as a developmental construct as it examines the quality of the child’s relational experiences from the child’s perspective (Baumrind, 1967; Epstein et al., 1983). It shifts away from describing relational health from caregiver behavior or the family climate and instead centers on the child’s experience of said safety, emotional attachment, and co-regulation (Miller et al., 2022).

Early relational health is thought to be crucial for childhood development and lifelong health and wellbeing (Belsky, 1984; Frosch et al., 2021; Hambrick et al., 2019). It is thought that these impacts occur in part via effects on the developing brain (Garner & Yogman, 2021a, 2021b; Schore, 2001; Willis & Eddy, 2022). The brain undergoes marked development and reorganization from gestation right through adolescence, with the period between conception and age three years marking a particularly rapid period of development. During this time, complex structural and functional brain changes result from molecular and cellular processes, including neurogenesis, neuronal migration, synapse formation, dendritic arborisation, axonal growth, pruning, and myelination (Ouyang et al., 2019). Such development renders the brain ‘plastic’ and hence sensitive to social environmental influences. Existing literature and reviews have examined the impact of relational health on various cognitive, psychopathological, and socioemotional outcomes (Frosch et al., 2021, Miller et al., 2022, Willis et al., 2022). Extending our understanding of the impact of early relational health on the developing brain is therefore important for understanding risk and resilience mechanisms for a range of life course outcomes.

Indeed, considerable animal research has shown that disruptions to the maternal–offspring bond can negatively impact the offspring’s social and cognitive skills via alterations in brain development (see review by Knudsen et al., 2006). There is evidence from human research that negative or traumatic infant-caregiving experiences (e.g., abuse and neglect) impair the development of the brain’s stress and emotion regulation mechanisms (Herzberg & Gunnar, 2020). However, the specific neural mechanisms are still not well understood. Further, the impact of other aspects of relational health such as positive relational experiences remains relatively understudied. We identified a single other review investigating the impact of relational health on neurodevelopment (Ilyka et al., 2021). However, the review narrowly focused on studies that examined the use of direct behavioral observations and excluded those solely based on self-report, which may not fully capture early relational health. It was also published four years ago. To obtain a more comprehensive and updated understanding of the association between early relational health and the developing brain, the present scoping review aimed to systematically synthesize the available cross-sectional and longitudinal evidence on associations between relational health (self-reported or observed) from birth to age three years, and brain structure, function, and connectivity across developmental periods (e.g., infancy, childhood, and adolescence, adulthood).

## Methods

### Literature Search

The present review was conducted in accordance with the Preferred Reporting Items for Systematic Reviews guidelines for scoping reviews (PRISMA-ScR) (Tricco et al., 2018). Literature searches were performed in the MEDLINE, PsycINFO, and Embase databases with no date restriction on August 14, 2023, and yielded 6960 articles. An updated search was performed on June 12, 2025, yielding 196 articles. See Supplementary Material (SM) for search algorithm used. The retrieved articles were then exported to the Living Review System (LRS; an artificial intelligence [AI]-assisted literature review management software; Grbin et al., 2022), and after duplicates were removed, there were 5048 articles for article screening. The LRS allows the use of AI to identify relevant/non-relevant articles and order articles by relevance during the title and abstract screening stage, and proposes a stopping point for screeners when the probability of all relevant articles having been screened is more than 90%. All articles were independently screened by two blinded study authors during the title and abstract and full-text screening stage. It should be noted that all articles were manually screened as the stopping point was never reached in the LRS. Disagreements in screening responses were resolved through discussion between the screeners and with the larger research team, reaching 100% agreement.

### Inclusion & Exclusion Criteria

In accordance with the Joanna Briggs Institute’s (JBI) Population/Concept/Context framework, we formulated the following inclusion criteria (Pollock et al., 2023).

#### Population

Studies with community samples of human infants from conception to age three (parents/caregivers with clinical symptoms were included if the child participant had no clinical diagnosis of physical or mental illness).

#### Concept

Examination of (1) infant relational health (i.e., measures of child or parent, caregiver, sibling, grandparent, peer behavior relevant to their relationship, or measures of these relationships) from birth to end of age 3 years, and (2) neurobiological markers (i.e., brain structure, function, and/or connectivity) and/or brain development (i.e., changes in brain structure, function, and/or connectivity) at any age.

#### Context

To enhance comprehensiveness of the review, we did not restrict our search to specific geographical, temporal, cultural, or social settings.

Studies were excluded if they were (i) not written in English; (ii) not human subjects; or (iii) a case study, review, meta-analysis, or not peer-reviewed. No date limits were applied.

### Data Extraction & Synthesis

Study characteristics and findings were extracted by one author into a standardized data extraction table (and checked by a second author). Extracted study data included the following: (i) country; (ii) design, sample characteristics (number of participants, mean child age at measurement of relational health and at imaging); (iii) the relational health measure (and how it was operationalised); (iv) imaging modality; and (v) relevant key findings. To aid synthesis of findings, studies were then grouped by relational health concept (e.g., maternal sensitivity, intrusiveness, attachment, parent–child interaction quality).

## Results

**Fig. 1:**
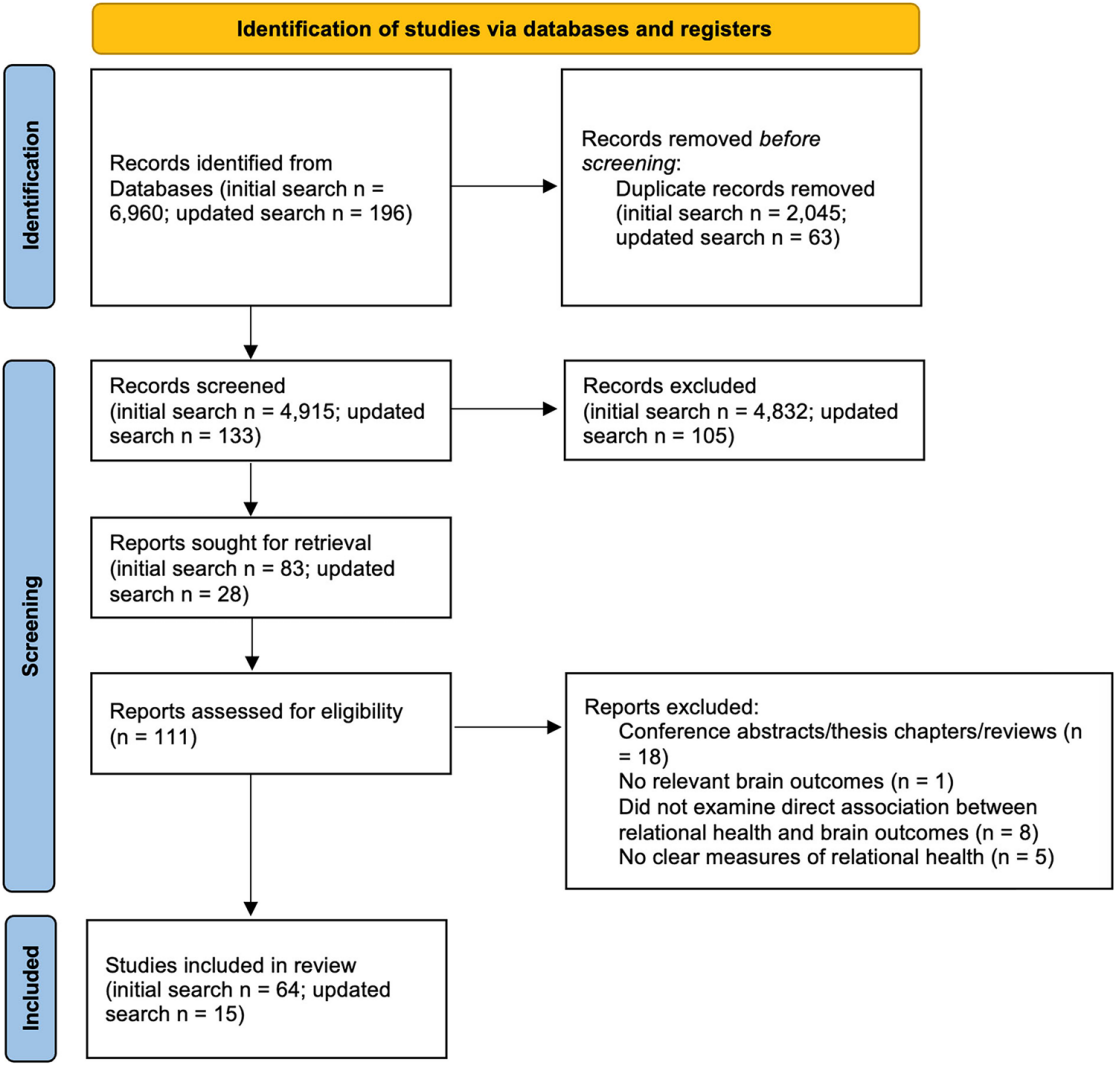
PRISMA diagram for article screening process. *Note*. Initial search was conducted on August 14, 2023 and updated search was conducted on June 12, 2025

The search resulted in 79 studies. See Fig. 1 for the number of articles included/excluded at each stage of the screening process and detailed exclusions. A large number (*n* = 42) employed electroencephalogram (EEG), while a smaller number of studies utilized structural magnetic resonance imaging (sMRI; *n* = 14), functional MRI (fMRI; *n* = 15; seven of which were resting-state studies), functional near-infrared spectroscopy (fNIRS; *n* = 8), and ultrasound (*n* = 1). Sample sizes ranged from 10 to 629 (average *n* = 102). Forty-one studies were cross-sectional (relational health and brain variables measured at the same timepoint), four were retrospective (brain assessments occurred prior to relational health assessments), and 34 were longitudinal. Of the longitudinal studies, the majority included one relational health assessment and one future neuroimaging assessment. Only eight had more than one brain imaging assessment, which may or may not have included an assessment concurrent with the relational health measure.

Most studies conducted neuroimaging during infancy/early childhood (*n* = 58). Only five studies conducted neuroimaging assessments during childhood (i.e., age 5–10 years), whereas 14 conducted neuroimaging assessments during adolescence/early adulthood. The oldest age at follow-up was young adulthood (22 years). Only two studies investigated father-infant relational health specifically (Kok et al., 2015; Sethna et al., 2019), and two studies included mothers and fathers but did not investigate relational health of these caregivers separately (Carver & Vaccaro, 2007; Sadeghi et al., 2019). All other studies investigated mothers only.

In terms of measures of relational health, the majority of studies (*n* = 34) assessed parent sensitivity, while a proportion investigated parent intrusiveness (*n* = 15), child-parent attachment (*n* = 16), and quality of parent–child interactions (*n* = 7). We structure our discussion of results by these relational health factors, further differentiating findings by developmental stage of neuroimaging outcomes (infancy/early childhood, [0–5 years], childhood [5–10 years], adolescence/early adulthood [10–24 years]).

### Sensitivity

#### Infancy/Early Childhood

Twenty-seven studies assessed parent sensitivity/responsiveness in relation to neurobiological measures in infancy/early childhood. Of these, all but one assessed maternal sensitivity (one assessed paternal sensitivity) and 17 employed EEG (two of which were longitudinal; Bernier et al., 2016; Frenkel et al., 2024). Pooling EEG study findings, greater maternal sensitivity was associated with relatively greater left frontal asymmetry in five studies (Field et al., 2003; Hane & Fox, 2006; Perone et al., 2020; Underwood & Gartstein, 2022; Wen et al., 2017), a pattern commonly interpreted as reflecting a tendency to experience more healthy, positive emotions and approach behavior. Supporting this, one study demonstrated that this asymmetry predicted subsequent lower negative emotionality (Wen et al., 2017). While three of these studies employed baseline EEG, two used negative emotion-eliciting tasks (Perone et al., 2020; Underwood & Gartstein, 2022). It may be that left frontal asymmetry in such tasks, instead of reflecting positive affect, reflects a healthy frustration response in infants when their typical exchanges with caregivers are marked by sensitivity (Underwood & Gartstein, 2022). These five studies were all cross-sectional. The one longitudinal study investigating frontal asymmetry found an effect of maternal sensitivity on later frontal asymmetry only in interaction with infant negative reactivity (Frenkel et al., 2024). Other cross-sectional studies reported null or contradictory findings. For example, Swider-Cios et al. (2024) found maternal sensitivity to be associated with more rightward frontal alpha asymmetry during free play but not the reunion phase of a still face paradigm.

Other EEG studies reported maternal sensitivity to be associated with lower baseline levels of frontal-posterior theta connectivity and higher frontal-posterior gamma connectivity (Perone & Gartstein, 2019), P100 latency (time delay between presentation of a visual stimuli and presence of the peak of the neural electrical signal [i.e., P100]) in the occipitotemporal region during eye gaze (The BASIS Team et al., 2015), increased negative central amplitudes to positive facial expressions (Taylor‐Colls & Pasco Fearon, 2015), less positive frontal P2 amplitudes (a pattern of neural electrical activity that occurs following visual/auditory stimuli) to angry than to happy utterances (Huffmeijer et al., 2020), and mother-frontal–infant-temporal neural synchrony (Endevelt-Shapira & Feldman, 2023). These findings may suggest that higher maternal sensitivity is associated with brain responses in infants that reflect an attentional or processing preference to positive stimuli, more mature patterns of brain development, and increased biological synchrony with mothers.

Fewer infant studies employed other neuroimaging techniques (i.e., sMRI, fMRI, and fNIRS). There were some inconsistencies in findings, such as positive (Sethna et al., 2017), negative (Rifkin-Graboi et al., 2015), and null (Sethna et al., 2019) associations between sensitivity and concurrent subcortical volumes. Other associations with brain volume/connectivity/activation and mother-infant brain synchrony were also noted (see Supplementary Material Table S1).

#### Childhood

Four studies investigated childhood neurobiological outcomes of parental sensitivity using MRI. In general, results support the continuing influence of parent sensitivity on brain structure and connectivity during mid-childhood. Parental sensitivity was associated with larger total brain volume and gray matter volume at 8 years, controlling for infant head size (Kok et al., 2015), and increasing subcortical volume (specifically, amygdala and basal ganglia volume) from age two to age seven (Treyvaud et al., 2021). Studies of functional connectivity suggest specificity of effects at different ages in terms of maternal sensitivity and connectivity measures. In one study, maternal sensitivity was associated with the right hippocampal functional connectivity with sensorimotor and top–down cognitive control networks at age four, whereas at six years of age, it was associated with hippocampal connectivity with a network involved in visual-processing (Wang et al., 2019). In another study, maternal sensitivity at 3 months (but not 30 months) predicted increased local connectivity within the medial prefrontal cortex at age five (Copeland et al., 2022), suggesting a sensitive period for the effects of parent sensitivity on offspring emotional and behavioral regulation. Of note, while three of these childhood studies investigated maternal sensitivity, one (Kok et al., 2015) investigated maternal and paternal sensitivity and found no differences in effects.

#### Adolescence/Early Adulthood

Only three studies investigated associations between maternal sensitivity and neurobiology in adolescents. Bernier et al. (2019) found maternal sensitivity to be associated with reduced hippocampal and amygdala volumes at age 10 (after controlling for attachment), similar to that reported by Rifkin-Graboi et al. (2015) in infancy. Valadez et al. (2020) found that 10-year-old children at risk of experiencing abuse and neglect, whose parents received an intervention to improve sensitivity (as compared to a comparison group), exhibited greater activation in response to images of their mother in regions supporting social-cognitive functions. This activity in turn was associated with reduced behavior problems. They also found that the intervention was associated with greater activation across fear and neutral faces in prefrontal and insula regions, and with negative connectivity between the amygdala and similar cortical regions (Valadez et al., 2024). The final study did not find associations between maternal sensitivity and brain activation in 20-year-olds during viewing of their own attachment videos (Ulmer-Yaniv et al., 2022).

### Intrusiveness

#### Infancy/Early Childhood

Fifteen studies assessed maternal intrusiveness or negative/harsh parenting in relation to neurobiological outcomes during infancy. Of these, ten studies employed EEG. In a pattern opposite to that found for sensitivity, cross-sectional findings revealed that infants of mothers with intrusive patterns of interacting showed greater relative right frontal asymmetry (during rest or exposure to novel stimuli) than infants that received good/high quality maternal caregiving (Field et al., 2003; Hane & Fox, 2006). Such findings may be interpreted as indicative of more negative reactive behavior (Hane & Fox, 2006) or withdrawal of motivated behavior (Jones et al., 1997). However, studies on infants of depressed mothers suggest that right frontal asymmetry is less apparent in infants of intrusive mothers than withdrawn mothers (Diego et al., 2006; Jones et al., 1997).

Longitudinal evidence also found a shift toward left frontal asymmetry from 3 to 6 months of age in infants of intrusive mothers, while infants of withdrawn mothers showed changes toward right frontal asymmetry (Diego et al., 2006). Such findings may reflect a possible compensatory neural mechanism to facilitate approach behavior in infants of intrusive depressed mothers and a lack of such in infants of withdrawn mothers (Jones et al., 1997); however, this is only speculative. Notably, one recent cross-sectional study also observed no association between maternal intrusiveness and infant frontal asymmetry (Diaz et al., 2019), highlighting a need for further research on this link between intrusive parenting and brain development during infancy.

Other EEG studies found that maternal intrusiveness at 5 months of age was associated with increased left midline brain region activation during attention tasks five months later (Swingler et al., 2017), but not with frontal activations at rest at 5, 10, and 24 months, or changes in activation during this developmental stage. One study found that harsh parenting at age two was not associated (at the group level) with error-related negativity during an attention task at age four (Brooker & Buss, 2014). However, in infants exposed to high levels of harsh parenting, increased fearfulness at age two was associated with increased error-related negativity at age four. This was interpreted by the authors as reflecting a possible exacerbation of the negative effect of fearfulness on the development of performance monitoring processes during late infancy. Finally, Endevelt-Shapira and Feldman (2023) found intrusiveness to be cross-sectionally associated with reduced mother-frontal–infant-temporal neural synchrony, a pattern opposite to that found for maternal sensitivity.

Using structural MRI, fNIRS, and resting-state functional connectivity, studies found possible associations between intrusiveness and reduced white matter volume (Lyons-Ruth et al., 2023), increased activation of temporal regions (as indicated by increased oxyhemoglobin level) to angry (relative to neutral) prosody (patterns of stress and intonation in a language; Zhao et al., 2019), and no association with network efficiency (Hanford, 2018). These findings may suggest alterations in the development of white matter connections, and processing of negative emotional stimuli among infants receiving high levels of maternal intrusiveness.

#### Childhood and Adolescence/Early Adulthood

Two studies examined early exposure to intrusive maternal parenting and later neural outcomes. Using sMRI, Treyvaud et al. (2021) found that intrusive parenting at age two was associated with smaller intracranial and total cortical volume, as well as lower fractional anisotropy and higher radial diffusivity in several white matter tracts (e.g., corticospinal tract, anterior thalamic ration) during childhood (i.e., age seven). Such findings may demonstrate a stunted growth in cortical gray matter and organization in white matter microstructure in these children (Gilmore et al., 2012; Mukherjee & McKinstry, 2006). Another study that examined outcomes in late adolescence found that harsh parenting at age two was associated with reduced right amygdala activity to fear stimuli, which was interpreted by the authors as a potential neural risk factor for antisocial behavior (Gard et al., 2017).

### Attachment

#### Infancy/Early Childhood

Eight studies assessed attachment status in relation to neurobiological outcomes during infancy, and six of these studies utilized EEG. Infants with disorganized attachments demonstrated a lack of right frontal asymmetry during exposure to distressing animations compared to those with other attachment styles (Biro et al., 2021). This may be interpreted as reflecting a lack of withdrawal tendencies and dysfunction in regulatory mechanisms to adaptively disengage from distressing stimulation.

For EEG activation, infants that displayed more proximity-seeking behaviors with their mother also displayed smaller and shorter latency Nc and P400 amplitude responses to their mother’s face relative to strangers’ (Swingler et al., 2007, 2010). These findings suggest that the infant’s attachment style impacts the neurophysiological attention that they direct to their primary caregiver over unfamiliar faces (Swingler et al., 2010).

Further, N290 amplitudes over the occipitotemporal region were more positive to fearful faces in secure infants (relative to non-fearful faces), but no significant results were revealed for infants classified as insecure in attachment status (Peltola et al., 2020). This might suggest a lack of distinction in the perceptual processing of facial emotions in insecure infants, which could have implications for the development of social competence. Null findings were also observed for attachment in relation to frontal and parietal activity in mother-infant play versus separation (Dawson et al., 1992).

Another study revealed that a larger gangliothalamic ovoid, measured using ultrasound six-weeks postpartum, predicted lower attachment disorganization scores when the infant was 14 months old (Tharner et al., 2011). This study was unique in this review by predicting attachment status through neurobiology, and findings were interpreted to suggest that smaller basal ganglia and thalamus immediately post-birth or even during gestation might be associated with impaired goal-directed attachment behavior. Finally, Minagawa et al. (2023), using fNIRS, found that more difficult attachment was associated with weaker synchronization of infant-temporal–mother temporal and frontal synchronization during breastfeeding.

#### Adolescence/Early Adulthood

All remaining studies utilized MRI during adolescence. Secure attachment was associated with larger gray matter volume in regions encompassing the temporal sulcus, gyrus, frontal gyri, and temporoparietal junction and was interpreted as preliminary evidence for potential impact on regions involved in social, cognitive, and emotional functioning (Leblanc et al., 2017). Associations with white matter microstructure (as measured by fractional anisotropy and radial diffusivity) were also found in a range of tracts including the corpus callosum, cingulum, and superior longitudinal fasciculus (Dégeilh et al., 2023); these authors suggest that this may reflect a positive influence of early secure attachment on optimal white matter development and in turn better cognitive abilities. On the other hand, disorganized attachment was associated with thicker cortices in frontal brain regions, including the orbitofrontal cortex, in early adolescence, which in turn was associated with greater peer rejection experiences (Leblanc et al., 2022).

Studies using fMRI generally support the finding that youth who were classified in insecure attachment relationships in infancy show greater later neural dysregulation than their peers who were securely attached in infancy. Compared to their securely attached counterparts, adolescents with early insecure attachment histories showed more activation in the dorsal lateral prefrontal cortex and anterior cingulate cortex extending into dorsal medial prefrontal cortex when viewing socially aversive cues (Rogers et al., 2022), as well as diminished sensitivity to risk in regions involved in reward processing, cognitive control, and salience detection, including the dorsal striatum, bilateral dorsal lateral prefrontal cortex, right ventral lateral prefrontal cortex, precuneus, and bilateral posterior insula (McCormick et al., 2019).

Individuals with early insecure attachment histories were found to exhibit hyperactivity in structures including the striatum, basal ganglia, amygdala, and cortical midline with reward-based tasks (McCormick et al., 2019; Quevedo et al., 2017). Further, young adults (age 22 years) with early insecure attachment histories displayed greater activation in prefrontal regions when attempting to up-regulate their positive emotions (Moutsiana et al., 2014). Together, these findings suggest that insecure attachment status in infancy may lead to enhanced processing of negative social information, enhanced sensitivity to rewarding outcomes combined with reduced sensitivity to potential risks, and greater effortful neural processing required to up-regulate positive emotions.

Two further studies implicated the hippocampus and amygdala in relation to attachment status. Moutsiana et al. (2015) found infants classified insecure in attachment to have greater amygdala volumes at age 22 years (relative to those securely attached as infants), and Cortes Hidalgo et al. (2019) found that infants with disorganized attachment status had larger hippocampal volume at 10 years of age compared to those with organized attachment styles. The authors interpreted these findings as potential indicators of accelerated brain development in response to the context of stressful experiences of care associated with infant disorganized attachment status (Cortes Hidalgo et al., 2019).

### Quality of Parent–Child Interactions

Seven studies investigated various qualities of the parent–child relationship, although methodologies varied considerably. Studies during infancy found support for higher quality parent–child interaction being associated with subsequent increases in absolute power of alpha and beta bands in the frontal lobe (Sadeghi et al., 2019), and lower theta power and higher alpha power cross-sectionally, and less growth (i.e., a less steep slope) in alpha power over time (Wade et al., 2024). Associations with greater dyadic alpha synchrony (Neel et al., 2025) and leftward frontal asymmetry (Neel et al., 2023) were also found during enhanced sensory scaffolding interactions. Additionally, functional connectivity within and between the salience and default mode networks during infancy was found to be associated with high parent–child quality (Li et al., 2023). Increased skin-to-skin contact was associated with greater EEG complexity in the right hemisphere during sleep in infants (Scher et al., 2009). Finally, in late adolescence, higher quality parent–child interaction was associated with larger total cerebral gray matter and caudate volume (Charpak et al., 2017).

### Other Relational Health Measures

Fourteen studies investigated other types of relational health but could not be grouped according to the aforementioned general themes. These studies investigated, for example, aspects of parent or infant behavior, such as parent mind-mindedness (Dégeilh et al., 2018), parent- or infant-led attention (Bagdasarov et al., 2025; Marriott-Haresign et al., 2023; Phillips et al., 2023; Williams et al., 2025), and infant affection (Dawson et al., 1999). See Supplementary Material Table S5 for further details.

## Discussion

Across all studies, two relatively consistent findings emerged. First, greater parent sensitivity and lower intrusiveness were associated with greater left frontal EEG asymmetry, a pattern widely regarded as reflecting greater positive or approach-related emotions (Palmiero & Piccardi, 2017), in addition to reduced risk for emotion dysregulation and associated mental health conditions such as depression (Allen & Reznik, 2015). These studies, however, were all conducted during infancy and early childhood, and the longer-term implications of these patterns of neural activity are unknown. Second, results supported the continuing influence of relational health on brain structure and connectivity during childhood and adolescence. Greater parental sensitivity and lower intrusiveness were associated with larger total brain volumes during adolescence and early adulthood, up to 20 years after relational health was assessed in infancy. Associations between relational health during infancy and other aspects of brain structure and function during childhood and adolescence were noted; however, there was limited consistency in findings, largely due to methodological differences between studies.

### Limitations

First, while many studies assessed neurobiology months or years after relational health was measured (and hence were technically longitudinal), very few conducted baseline neuroimaging. As such, interpretation of impacts of relational health on brain development should be taken with caution. Investigations of brain change over time (i.e., brain development) during periods of rapid brain maturation are critical for understanding mechanisms of social environmental impacts on health and wellbeing outcomes (Becht & Mills, 2020). Further, a lack of longitudinal studies with repeat investigations of relational health and the brain mean that the direction of findings is unclear. It is possible that there are bi-directional association between early relational health and neurobiology. For example, brain development (during gestation or later) may affect relational health.

The majority of studies included investigated only mothers, with just a few including fathers and no studies investigating the other relationships with and around the child that constitute early relational health. As such, our current understanding of the impact of relational health on brain development is limited to health of the mother–child relationship.

While not a limitation per se, it is of note that the LRS did not reach a stopping point, and thus, all papers were screened manually. The LRS has been shown to be highly accurate and reliable, with 22–53% of articles typically requiring human screening (Fuller-Tyszkiewicz et al., 2025). We speculate that no stopping point was reached for this review due to the highly heterogeneous nature of the brain imaging methods utilized across studies.

### Future Directions

Based on findings from the present review, there is evidence that early relational health may have impacts on the brain that extend well beyond childhood into adolescence and young adulthood. However, we also note that research in this area is still in its infancy. Based on the available evidence, we make the following suggestions for future research efforts.

#### Actionable Insights

- Longitudinal research is needed to investigate the impacts of relational health on brain development trajectories. Such research should employ consistent methodology to assess brain structure and function over time.
- Randomized-control-trials or observational studies employing causal mediation analysis techniques are needed to confirm the directional and causal nature of associations between early relational health and brain development.
- Research is needed that is more comprehensive and holistic in the investigation of relational health, including the involvement of fathers, other caregivers, and interactions with broader social factors.
- Research is needed to investigate the significance of relational-health-associated neurobiology for health and wellbeing. This may include employing causal mediation analysis and investigation of health and wellbeing outcomes.

## Conclusion

Patterns of early relational health (both secure and insecure), from conception to three, are associated with brain structure, function, and connectivity from infancy to young adulthood. Specifically, higher parent sensitivity and lower intrusiveness, and secure infant-parent attachment status are associated with patterns of brain activity that are likely beneficial for future mental health and wellbeing. However, few studies have employed longitudinal designs that allow the examination of change in relational health and brain structure/function/connectivity over time, limiting the ability to draw conclusions regarding the association between early relational health and dynamic brain development. Further, few studies have examined relational health beyond the mother–infant relationship, leaving the influence of a large proportion of other types of relational health understudied. Future research addressing these key gaps in the literature is therefore warranted.

## Supplementary Information

Below is the link to the electronic supplementary material.

Supplementary file1 (DOCX 75 KB)
