# Supplementary material for: Early Relational Health and its Impact on the Developing Brain: A Scoping Review
Source: Clin Child Fam Psychol Rev. 2025 Sep 17;29(2):229–40. doi: 10.1007/s10567-025-00545-3 (PMC13282346; doi:10.1007/s10567-025-00545-3)
Supplement: Supplementary file 1 — Supplementary file1 (DOCX 75 KB) [file 10567_2025_545_MOESM1_ESM.docx]

Supplementary Information

**Table 1**

Study characteristics of included studies measuring maternal sensitivity (those who also measured intrusiveness are described here)

| **Study** | **Country** | **Design** | **N** | **Predictor measure** | **Predictor period** | **Imaging modality** | **Outcome period** | **Key Findings** |
| --- | --- | --- | --- | --- | --- | --- | --- | --- |
| Bernier et al.(2016) | US | LO | 197 (105 female) | 2-minute toy play, coded for maternal interactive style: sensitivity, intrusiveness, positive affect, physical stimulation (Calkins, Hungerford, & Dedmon 2004) | 162 days (8)  (i.e., 5 months) | EEG (baseline) | 5, 10, and 24 months | Higher-quality maternal behaviour (more positive affect and less physical stimulation) during mother-infant interactions predicted higher frontal resting EEG power at 10 and 24 months, as well as increases in power between 5 and 10 months, and between 10 and 24 months. Sensitivity/Intrusion was unrelated to infant EEG. |
| Wen et al. (2017) | Singapore | CS | 111 (62 female) | 15-minute mother-child interaction assessed using Mini-MBQS-V | 26.39 weeks (0.95) | EEG (during passive auditory oddball task) | 26.39 weeks (0.95) | In mothers who spend >50% time with infant, lower maternal sensitivity was associated with greater relative right frontal EEG asymmetry. |
| Perone et al. (2020) | US | CS | 10 (3 female) | SFP | 8–12 months, 10.72 | EEG (during SFP) | 8–12 months, 10.72 | Only descriptive results. Results showed dyads exhibited a rightward shift in frontal alpha asymmetry. Dyads with a more responsive mother exhibited higher (more left) asymmetry relative to dyads with a less responsive mother. |
|  |  |  |  |  |  |  |  |  |
| Underwood & Gartstein (2022) | US | CS | 53 (24 female) | 2-minute mother-infant free-play coded to reflect interaction dynamics | 37.08 weeks (7.83) | EEG (during toy retraction) | 37.08 weeks (7.83) | Sensitivity/responsiveness was associated with significantly greater activation (i.e. less alpha power) in the left hemisphere |
| Hane & Fox (2006) | US | CS | 185 | Behaviour coded during visit using a modified version of Ainsworth’s (1976) ratings for sensitivity and intrusiveness | 9 months | EEG (baseline) | 9 months | Those in the low-quality MCB group (low sensitivity/high intrusiveness) were significantly more likely to exhibit a pattern of right frontal EEG asymmetry |
| Field et al. (2003) | US | RE | 140 | Interaction coded for intrusive, withdrawn, or "good interaction" | 3 months | EEG (baseline) | 1.9 days | The “good interaction” depressed group as compared to the withdrawn group had less relative right frontal EEG activation in the newborns; and the “good interaction” depressed group of newborns as compared to the intrusive group had less relative right frontal EEG activation. |
| Perone & Gartstein (2019) | US | CS | 51 | 2-minute free play mother-infant interaction session scored for responsiveness, reciprocity, and emotional tone | 35.57 weeks (1.11) | EEG (baseline) | 35.57 weeks (1.11) | Frontal-posterior theta connectivity was inversely related to responsiveness, reciprocity, and emotional tone. Frontal-posterior alpha connectivity was positively related to emotional tone only. Frontal-posterior beta connectivity was unrelated to parent-infant interactions. Gamma was positively related only to responsiveness. |
| Elsabbagh et al. (2015) | UK | CS | 93 (55 female) | 6-minute free play coded using MACI | 238.3 days (37.2) | EEG | 238.3 days (37.2) | There was a significant main effect of parent sensitive responsiveness on the P100 latency difference in the control group. |
| Taylor-Colls & Pasco Fearon (2015) | London | CS | 40 (17 female) | EAS | 230 days (9.8) | EEG, ERP | 230 days (9.8) | There was a significant Emotion x Sensitivity interaction. Higher maternal sensitivity was associated with a larger, more negative, Nc for happy faces, relative to neutral faces. |
| Huffmeijer & Gervain (2020) | France | LO | 22 (11 female) | Videotaped 15 minute free-play session coded for sensitivity and intrusiveness (Ainsworth) | 24–28 weeks, 25.95 (1.21) | EEG | 28–36 weeks, 30.82 (1.79) | Significant interaction effect of condition and maternal intrusiveness on infants’ P2 amplitudes. Maternal intrusiveness was related to the difference in infants’ ERP responses to happy and angry utterances. |
| Gartstein (2020) | US | CS | 59 (34 female) | Videotaped 2 minutes of free play coded for sensitivity, reciprocity, tempo, intensity, and emotional tone | 6–12 months, 8.44 (1.51) | EEG (during SFP) | 6–12 months, 8.44 (1.51) | Two significant main effects for parent–child interaction factors – intensity and tone were predictive of relative right frontal activation. No significance for sensitivity |
| Swingler et al. (2014) | US | CS | 233 (125 female) | Interaction observed during orientation and distraction while infant arm restrained; maternal sensitivity observed during two mother-child tasks | 162 days (8) | EEG (baseline) | 162 days (8) | EEG frontal asymmetry is not associated with maternal sensitivity, orientation, distraction. |
| Gartstein, et al. (2021) | US | CS | 59 (34 female) | 2-minute mother-infant free-play coded for responsiveness, reciprocity/synchrony, intensity, and directedness | 8.44 months (1.51) | EEG (during SFP) | 8.44 months (1.51) | Infants who shifted toward stronger frontal activation of the left hemisphere experienced greater reciprocity when interacting with mothers. Mother–infant interactions among infants shifting toward greater activation on the right were more directive and intense. |
| Endevelt-Shapira & Feldman (2023) | Israel | CS | 60 (25 female) | 3-minute mother-infant free-play coded for sensitivity and intrusiveness using the Coding Interactive Behavior Manual | 4-12 months, 6.95 (1.42) | EEG (during free play) | 4-12 months, 6.95 (1.42) | Mother-frontal–infant-temporal neural synchrony was positively correlated with maternal sensitivity and negatively correlated with maternal intrusiveness. |
| Frenkel et al. (2024) | US | LO | 51 (20 female) | From 3-minute mother-infant free-play. maternal contingent responsiveness calculated as the relative amount of infant gazes toward mother that were followed, within 2 s, by maternal gaze, and/or maternal positive affect, and/or maternal motherese vocalization toward her infant. | 3-5 months, 4.17 (0.34) | EEG (baseline) | 8-15 months, 10.9 (1.14) | No direct association between maternal responsiveness and EEG alpha frontal asymmetry. Infant negative reactivity predicted relative left infant frontal EEG asymmetry only at low levels of maternal responsiveness. |
| Swider-Cios et al. (2024) | Netherlands | CS | 36 (19 female) | SFP | 5-12 months, 9.16 (1.3) | EEG | 5-12 months, 9.16 (1.3) | Significant association between maternal affective sensitivity and more rightward (negative) frontal alpha asymmetry scores in infants during baseline (free play) (not significant for reunion). |
| Sethna et al. (2017) | UK | CS | 43 (21 female) | 5-minute play coded using the Global Rating Scales (dimensions of sensitivity, affect, communication and fretfulness) | 4.83 months (1.15) | MRI | 4.83 months (1.15) | Greater infant communication and engagement during mother–infant interactions was associated with smaller cerebellum volumes. Infants interacting with less sensitive mothers had smaller subcortical grey volumes. |
| Rifkin-Graboi et al. (2015) | Singapore | CS | 32 | Mini-MBQS-V | 6 months (± 2 weeks) | sMRI, rsfMRI | 6 months (± 2 weeks) | Maternal sensitivity significantly predicted left and right hippocampal volume at 6 months. Maternal sensitivity significantly positively predicted functional connectivity between the right hippocampus and bilateral ventromedial prefrontal cortex, right dorsolateral pre-frontal cortex, left fusiform and right middle temporal cortex. Maternal sensitivity positively predicted left hippocampal connectivity to left fusiform, left superior temporal cortex, left lateral occipital cortex. Maternal sensitivity negatively predicted connectivity with right lingual gyrus and right posterior cingulate, as well as left hippocampal connectivity, with the left entorhinal cortex. Maternal sensitivity negatively predicted right amygdala connectivity with the left inferior temporal cortex, and negatively predicted left amygdala connectivity with the left entorhinal cortex and left middle temporal cortex. |
| Sethna et al. (2019) | UK | CS | 28 (16 female) | 5-minute play coded using the Global Rating Scales (focus on sensitivity dimension) | 144 days (30) | MRI | 144 days (30) | Paternal sensitivity was not significantly associated with subcortical grey matter volumes. Paternal sensitivity was negatively associated with cerebellar volume, with a large effect size. Smaller cerebellar volumes were observed in more communicative and engaged infants who are also exposed to more sensitive paternal behaviours. |
| Chajes et al. (2022) | US | CS | 50 (19 female) | 5-minute free play, coded using sensitivity and cooperation (Ainsworth’s (1969) Sensitivity Scales) | 4–7 months, 5.2 (0.68) | fNIRS | 4–7 months, 5.2 (0.68) | Maternal sensitivity composite was significantly associated with greater infant DMN connectivity. |
| Mateus et al. (2021) | Portugal | LO | 24 (11 female) | 3 mother-infant interactions coded using Maternal Sensitivity Scales (Ainsworth) | 7.62 months (0.37) | NIRS probe | 12.89 months (0.31) | For the channels placed over the somatosensory cortex, infants whose mothers were less sensitive tended to present a higher response peak in HbO2 to the affective touch condition. For channels centered over the temporal area, less sensitive maternal behaviour was significantly associated with infants’ higher peak amplitude of HbO2. |
| Stern et al. (2024) | US | LO | 106 (45 female) | Mother-infant free play coded using Maternal Sensitivity Scales (Ainsworth) | 5 months | fNIRS | 5 & 7 months | Maternal sensitivity was significantly associated with infants’ greater dlPFC response in the happy face condition (but not the fear or angry condition), and relative increases in infant dlPFC responses to happy faces from 5 to 7 months. |
| Minagawa et al. (2023) | Japan | CS | 71 (26 female) | SFP – coded using sing ELAN (Brugman and Russel, 2004) | 122.4 (16) days | fNIRS | 122.4 (16) days | There were no correlations between brain synchrony (wavelet transforms coherence) and the mother’s contingent responsiveness during free play. |
| Treyvaud et al. (2021) | Australia | LO | 118 (57 female) | 10-minute structured play, coded for sensitive and intrusive parenting behaviours | 2 years | MRI | 7 years | More sensitive parenting at 2 years was associated with larger basal ganglia volumes at 7 years for boys, greater growth in basal ganglia volume from term to 7 years, and greater growth in amygdala volume growth for boys. More intrusive parenting was associated with smaller intracranial and grey matter volumes at 7 years, as well as lower fractional anisotropy and higher radial diffusivity in the cerebellar white matter, cerebral peduncle, corticospinal tract, anterior thalamic radiation, and superior longitudinal fasciculus at 7. |
| Rahkonen et al. (2014) | Finland | CS | 64 (22 female) | 15-minute mother-child structured play, coded using Erickson Scales and MRO | 2 years | MRI | 2 years | Neurological impairments at two years, white matter or gray matter abnormalities in MRI at term-equivalent age, and grade III-IV intraventricular haemorrhage during the neonatal period were not associated with mother-child interaction. |
| Kok, 2015 | Netherlands | LO | 191 | Age 1: 5 minute free-play session between child-parent dyads;  Age 3: 3–4-minute parent-child difficult task (puzzle/etch-a-sketch) completed with primary carer;  Age 4: 3–4-minute parent-child difficult task (puzzle/etch-a-sketch) completed with both parents;  Both 3/4 timepoints coded using rating scales for supportive presence and intrusiveness | 1,3, and 4 years | MRI | 8.04 years (0.93) | Higher parental sensitivity/low intrusiveness in early childhood were associated with larger total brain volume and gray matter volume at 8 years. Higher maternal sensitivity/low intrusiveness in early childhood were associated with a larger gray matter volume at 8 years. Associations with maternal versus paternal sensitivity/intrusiveness were not significantly different. |
| Wang et al. (2019) | Singapore | LO | Age 4: 61 (33 female)  Age 6: 76 (46 female) | Mini-MBQS | 6 months ± 2 weeks | rs-fMRI | Age 4: 4.36–4.75 years, 4.57 (0.08)  Age 6: 5.83–6.61 years, 6.03 (0.13) | At four years of age: Maternal sensitivity during infancy was positively associated with 4-year-old’s functional connectivity between the right aHPC and the right precentral gyrus, the left postcentral gyrus, and the right postcentral gyrus. Maternal sensitivity was negatively associated with functional connectivity between the right aHPC and the left dorsolateral prefrontal cortex (dlPFC).  At six years of age: Maternal sensitivity during infancy was positively associated with the right aHPC functional connectivity and aspects of the visual-processing network, including the left calcarine, right calcarine, right lingual, and left cuneus cortex. |
| Copeland et al. (2022) | Finland | LO | 17 (11 female)  39 (26 female) | EAS | Infant: 7.4–8.7 months,8.0 (0.4)  Toddler: 29.5–31.4 months, 30.1 (0.4) | rs-fMRI | 5.3–5.8 years, 5.4 (0.1) | Infants: Among the 17 mother–child dyads, the multiple regression analysis for ReHo and maternal sensitivity during infancy showed a positive association with the mPFC. Peak cluster was located in the left medial superior frontal gyrus with extensions to the right medial superior frontal gyrus and right anterior cingulate and paracingulate gyri. There was a significant positive partial correlation between mean ReHo values and maternal sensitivity scores.  Toddlers: Analyses of the 39 mother–toddler dyads showed no statistically significant associations between maternal sensitivity during toddlerhood and ReHo maps at the age of 5 years. |
| Bernier et al. (2019) | Canada | LO | 33 (20 female) | MBQS | 11.0–16.5 months, 13.02 (1.32) | sMRI | 10.0–11.67 years, 10.59 (0.46) | Dimension maternal sensitivity = The three dimensions of sensitivity together predicted volume of the left hippocampus. This prediction was mostly due to Positivity, which negatively predicted left hippocampal volume. |
| Valadez et al. (2020) | US | LO | 68 | 10-session home-based intervention, targets of "increasing sensitivity to child signals, increasing nurturance to child distress, and decreasing ... harsh behaviors" | Infancy | fMRI | 10.0 years (0.8) | Children whose parents received the ABC intervention exhibited greater activation in response to mother (compared with stranger) images than children whose parents received the DEF intervention. These effects were observed in clusters including the precuneus and cuneal cortex, PCC, middle temporal gyrus, lateral occipital cortex, angular gyrus, and hippocampus. |
| Valadez et al. (2024) | US | LO | 60 | 10-session home-based intervention, targets of "increasing sensitivity to child signals, increasing nurturance to child distress, and decreasing ... harsh behaviors" | Infancy | fMRI | 10.1 years (0.8) | Children whose parents received the attachment and biobehavioral catch-up intervention exhibited greater activation across fear and neutral faces than the control intervention group (i.e., developmental education) in clusters of brain regions including the anterior cingulate cortex, right orbitofrontal cortex, and right insula. They also showed negative connectivity between the amygdala seed and a cluster of brain regions including the right insula and right frontal orbital cortex (whereas the control group showed positive connectivity). |
| Ulmer-Yaniv et al. (2022) | Israel | LO | 65 (32 female) | T1: 5-minute mother-infant free-play; T2 and T3: mother-adolescent engaged in 7-minute conversation-based positive interaction coded using CIB | T1: 4.8 months (1.1)  T2: 10.9 years (1.2)  T3: 20.03 years (2.0) | fMRI | 20.03 years (2.0) | Self-related attachment videos elicited stronger BOLD activity, and this difference was significant in all regions except for the DMN. Significant self-related attachment effects were found in the insula, temporal cortex, and amygdala. ACC beta values for the Self condition were not correlated with maternal sensitivity. |
|  |  |  |  |  |  |  |  |  |

**Table 2**

Study characteristics of included studies measuring intrusiveness

| **Study** | **Country** | **Design** | **N** | **Predictor measure** | **Predictor period** | **Imaging modality** | **Outcome period** | **Key Findings** |
| --- | --- | --- | --- | --- | --- | --- | --- | --- |
| Jones et al. (1997) | US | CS | 140 | 3-minute interaction coded as intrusive, withdrawn, "good-interaction" | 3 months | EEG | 3 months | The depressed mothers and their newborns also had greater relative right frontal EEG activation. The “good interaction” depressed group as compared to the withdrawn group had less relative right frontal EEG activation in both the mothers and newborns; and the “good interaction” depressed group of newborns as compared to the intrusive group had less relative right frontal EEG activation. |
| Diego et al. (2006) | US | LO | 66 (45 female) | 3-minute mother-infant free-play, coded for depressed, non-depressed, intrusive, or withdrawn | Neonatal: 0–3 weeks, 1.7 (0.79)  Infant: 12–23 weeks, 17 (3.3) | EEG | Neonatal: 0–3 weeks, 1.7 (0.79)  Infant: 12–23 weeks, 17 (3.3) | Infants of depressed withdrawn mothers exhibited greater relative right frontal EEG activation than infants of depressed intrusive mothers. Furthermore, while infants of depressed mothers with intrusive interaction styles showed a shift towards greater relative left frontal EEG activation from birth to 3–6 months, infants of depressed mothers with withdrawn interaction styles showed a shift towards greater relative right frontal EEG activation. |
| Diaz et al. (2019) | US | CS | 410 (209 female) | 5 months: Mother-infant 2 minute free-play interaction  24 months: Mother-child 5-minute puzzle interaction | 5 and 24 months | EEG (baseline) | 5 months | No significant correlations. |
| Swingler et al. (2017) | US | LO | 388 (199 female) | Play tasks coded as maternal positive affect and maternal intrusiveness | 5 months | EEG | 5 months, 10 months | Maternal intrusiveness at 5 months was associated positively with infant’s 10-month baseline to attention task change in EEG power values at the left frontal midline location. In contrast, intrusiveness was not associated with infants’ baseline to attention task change in power values at the right medial frontal location. Intrusiveness was not associated with frontal activations at rest at 5, 10, and 24 months. |
| Brooker & Buss (2014) | US | LO | 41 (20 female) | Parent response to two questionnaires assessed with CNES (modified for infants); vignette assessing critical control, appropriate support, overprotection | 2.04 years (0.04) | EEG | 4.59 years (0.13) | Greater age 2 fearfulness predicted greater ERN at age 4. In contrast, at low levels of harsh parenting during toddlerhood, greater age 2 fearfulness marginally predicted smaller (i.e., less negative) ERN at age 4. High levels of fearfulness at age 2 greater harsh parenting were associated with slower response times. In contrast, at low levels of age 2 fearfulness, harsh parenting was unrelated to response times. |
| Zhao et al. (2019) | UK | CS | 29 (15 female) | 6-min free-play coded using MACI subscales: sensitivity, directiveness (reverse coded for 'nondirectiveness') | 175–214 days, 189 (9.66) | fNIRS | 175–214 days, 189 (9.66) | Although HbO2 changes in neither region were associated with maternal sensitive responsiveness, increased activation to angry minus neutral prosody was negatively correlated with maternal directiveness. |
| Lyons-Ruth et al. (2023) | US | LO | 57 (29 female) | SFP – coded using AMBIANCE (Lyons-Ruth et al., 1999) | 4 months | MRI | 4–24 months, 12.28 (5.99) | Infant whole brain GMV was negatively associated with maternal withdrawal at 4 months. Maternal withdrawal accounted for 4.53% of adjusted GMV. Infant right hippocampal volume was negatively associated with maternal withdrawal, but only at older ages. Infant WMV was reduced in relation to increased maternal negative/inappropriate interaction. Maternal negative/inappropriate interaction accounted for 12.46% of adjusted infant WMV. |
| Hanford et al. (2018) | US | CS | 46 (22 female) | 5-min interaction coded using CITMI-R dimensions of hostility, intrusiveness, involvement, warmth, and sensitivity | 3.3 months (0.8) | rs-fMRI | 3.3 months (0.8) | While relationships between infant emotional behaviour or maternal caregiving, and nodal metrics were weak, higher levels of maternal MST strengthened associations between infant positive emotionality and nodal metrics within prefrontal, and occipital cortices more generally. Positive and negative aspects of maternal caregiving had little effect. |
| Gard et al. (2017) | US | LO | 310 (all male) | Observation and interview using Acceptance scale from HOME | 2 years | fMRI | 20 years | Harsh parenting in toddlerhood uniquely predicted lower right amygdala reactivity to fearful facial expressions in adulthood. |

**Table 3**

Study characteristics of included studies measuring attachment

| **Study** | **Country** | **Design** | **N** | **Predictor measure** | **Predictor period** | **Imaging modality** | **Outcome period** | **Key Findings** |
| --- | --- | --- | --- | --- | --- | --- | --- | --- |
| Swingler et al. (2010) | US | CS | 30 (14 female) | Infant shown stimuli of mother or stranger face; Mother-infant behavioural session (7 intervals of separation and reunions with mother) | 183.4 days | EEG | 183.4 days | No significant clusters were found when comparing neural oscillatory activity in the time before infant led and adult led mutual attention episodes. Likewise, there were no significant differences observed when comparing infant led mutual attention episodes and infant led nonmutual attention episodes. |
| Biro et al. (2021) | Netherlands | RE | 130 | SSP | 376.60 days (13.03) | EEG | 309.53 days (13.30) | Only insecure-avoidant infants responded with a significant decrease of right-sided frontal asymmetry during the Response Segment compared to the Separation Segment. During the Separation Section, both insecure-avoidant, and insecure-resistant infants differed from the disorganized group, while secure infants did not. During the Response Segment on the other hand, secure, and insecure-resistant, but not insecure–avoidant infants differed from disorganized infants. |
| Peltola et al. (2020) | Finland | RE | 61 | SSP | 415 days (21.13) | EEG | 213 days (2.96) | The N290 amplitudes were more positive to fearful than to non-fearful faces in securely attached infants. In insecurely attached infants, no significant main effect of Emotion was observed. For the P400 amplitudes, the Emotion x Attachment interaction was not significant at the nominal alpha level. |
| Swingler et al. (2007) | US | LO | 30 (14 female) | Infant behaviour coded throughout introduction of an unfamiliar adult, and two separations and reunions with the mother | 173–191 days, 183.4 | EEG | 173–191 days, 183.4 | Nc amplitudes resulting from viewing the stranger’s face were significantly more negative than were amplitudes resulting from viewing the mother’s face. More mother-directed proximity-seeking behaviours were associated with larger Nc amplitudes when viewing the stranger’s face, in comparison to viewing the mother’s face. |
| Dawson et al. (1992) | US | LO | 26 | SSP | 11–17 months, 14.21 (1.27) | EEG | 11-17 months | No significant observed for attachment in relation to frontal and parietal activity in mother-infant play versus separation. |
| Tharner et al. (2011) | Netherlands | RE | 629 (315 female) | SSP | 14.7 months (0.9) | Ultrasound | 6.75 weeks (1.77) | A larger gangliothalamic ovoid diameter 6 weeks postpartum predicted a lower attachment disorganization score at 14 months (similar for both genders and hemispheres). A larger diameter of the gangliothalamic ovoid (per 1-SD increase in diameter) predicted a 25% lower risk of a disorganized attachment classification. |
| Leblanc et al. (2017) | Canada | LO | 33 | AQS | 14.50 – 18.00 months, 15.65 (0.97) | MRI | 10.0–11.67 years, 10.59 (0.46) | Children who were more securely attached to their mother in infancy had larger grey matter volumes in the right hemisphere covering the superior temporal sulcus and gyrus, extending to the middle temporal gyrus, and into the temporo-parietal junction. Increased grey matter volume in the left superior temporal sulcus and in the bilateral precentral gyri was also related to higher attachment quality. Attachment security in infancy was not significantly related to cortical thickness in late childhood, neither positively nor negatively. |
| Leblanc et al. (2022) | Canada | LO | 33 | SSP | 18.1 months (0.8) | MRI | 10.59 years (0.46) | Results indicated that disorganized attachment was not associated with grey matter volumes. However, children who exhibited more disorganized attachment behaviours in infancy had significantly thicker cortices in bilateral middle and superior frontal gyri, and extending to the inferior frontal gyrus, as well as the orbitofrontal and insular cortices in the right hemisphere in late childhood. Moreover, children with thicker cortices in these regions experienced greater peer rejection, as rated by themselves and their teachers. |
| Dégeilh et al. (2023) | Canada | LO | 32 (20 female) | AQS (average of T1 and T2) | T1: 14.50–18.0 months,15.68 (0.96)  T2: 25.0–27.57 months, 26.40 (0.76) | MRI | 10.0–11.7 years, 10.61 (0.46) | More secure mother-child attachment was associated with significantly lower fractional anisotrop and higher radial diffusivity in the bilateral corpus callosum (genu and body), cingulum, corticospinal tract and the left superior longitudinal fasciculus. In addition, more secure mother-child attachment was associated with significantly lower fractional anisotrop in the right superior longitudinal fasciculus, bilateral inferior fronto-occipital fasciculus and right cerebral peduncle. |
| Rogers et al. (2022) | US | LO | 51 (17 female) | Modified SSP | 31–35 months, 32.61 (0.76) | fMRI | 13.2 years (0.56) | Secure adolescents showed fewer false alarms to aversive cues than to control cues, whereas this association was nonsignificant for insecure adolescents, indicating that secure (but not insecure) adolescents demonstrated better behaviorual regulation to aversive cues when alone. In the parental presence condition, insecure adolescents showed fewer false alarms to aversive cues than to control cues, and this association was nonsignificant for secure adolescents. |
| Quevedo et al. (2017) | US | LO | 171 (100% male) | SSP | 18 months | fMRI | 20 years | Results indicated that individuals with a history of insecure attachment showed hyperactivity in reward- and emotion-related (e.g., basal ganglia and amygdala) structures and emotion regulation and self-referential processing (cortical midline structures) in response to positive and negative outcomes (and anticipation of those outcomes). Further, the neural activation of individuals with a history of disorganized attachment suggested that they had greater emotional reactivity in anticipation of reward and employed greater cognitive control when negative outcomes were encountered. |
| McCormick et al. (2019) | US | LO | 50 (16 female) | Modified SSP | 2.5 months | fMRI | 12.50–14.83 years, 13.27 (0.61) | Relative to adolescents with secure attachment histories, adolescents with insecure histories showed blunted tracking of risk in the bilateral dorsal striatum, bilateral DLPFC, right VLPFC, precuneus, and bilateral posterior insula, regions involved in reward processing, cognitive control and salience detection, respectively. In contrast to results examining risk, adolescents in the insecure group showed enhanced tracking of increasing reward value in the bilateral dorsal striatum, bilateral anterior insula, anterior cingulate cortex, and left VLPFC. |
| Moutsiana et al. (2015) | UK | LO | 59 (29 female) | SSP | 18 months | sMRI | 22.4 years (0.65) | With equivalent analysis, significant main effect of infant attachment. Greater amygdala volumes were found in adults who had been insecurely attached infants relative to their securely attached counterparts. Significant positive associations were observed for maternal total months of depression, but there were no other significant effects. |
| Cortes Hidalgo et al. (2019) | Netherlands | LO | 551 | SSP | 14.6 months (0.9) | MRI | 10.15 (0.6) years | Children with disorganized infant attachment had larger hippocampal volumes than those with organized attachment patterns. Disorganized attachment did not predict any other difference in brain morphology. |
| Moutsiana et al. (2014) | UK | LO | 54 (24 female) | SSP | 18 months | fMRI | 22.4 years (0.65) | While attempting to up-regulate positive emotions, adults who had been insecurely versus securely attached as infants showed greater activation in prefrontal regions involved in cognitive control and reduced co-activation of nucleus accumbens with prefrontal cortex. Significant differences were identified in four regions; left and right anterior PFC/ frontal pole, rostral ACC (rACC), and dorsal medial prefrontal cortex (dmPFC). In each case, greater activation was observed in the insecure than the secure group during up-regulation relative to passive viewing of positive pictures. No significant findings for NAcc |
| Minagawa et al. (2023) | Japan | CS | 71 (26 female) | Mother-infant attachment (Postpartum Bonding Questionnaire) | 122.4 (16) days | fNIRS | 122.4 (16) days | Two synchronizations between the infant’s left superior temporal gyrus and the mother’s left superior temporal gyrus or left orbitofrontal cortex during breastfeeding showed a negative correlation, indicating more difficult attachment associated with weaker synchronization. |

**Table 4**

Study characteristics of included studies measuring qualities of parent-child interactions

| **Study** | **Country** | **Design** | **N** | **Predictor measure** | **Predictor period** | **Imaging modality** | **Outcome period** | **Key Findings** |
| --- | --- | --- | --- | --- | --- | --- | --- | --- |
| Sadeghi et al. (2019) | Iran | LO | 12 | Child-parent relationship scale - short form; Parent-infants completed two-month training intervention (more intentional hours together, more communication between people, less repetitive activities) | 24-47 months, 33.33 (9.95) | EEG | Approx. 4 months later | Significant increases were found in absolute power of alpha and beta frequency bands following the intervention. |
| Scher et al. (2009) | US | CS | 134 | Each child received one and one-half hours of skin-to-skin contact, four days a week, for eight weeks | Birth to 8 weeks | EEG | Birth to 8 weeks | Five linear measures distinguish the SSC pilot group from the two non-SSC cohorts and consist of fewer REMs, longer sleep cycle lengths, higher percentage of quiet sleep, less spectral beta power, and increased spectral respiratory irregularity. |
| Neel et al. (2025) | US | CS | 47 (24 female) | Structured naturalistic interaction in which the mother provided infant-directed sensory stimuli. Welch Emotional Connection Scale used as an indicator of mother–infant connection/responsiveness. | 94 days (median 78, IQR 114) | EEG | 94 days (median 78, IQR 114) | Greater mother-infant connection/responsiveness was associated with greater increases in dyadic alpha synchrony with enhanced sensory scaffolding. |
| Neel et al. (2023) | US | CS | 51 (25 female) | Structured naturalistic interaction in which mother provides progressive increase in number of sensory modalities.  Welch Emotional Connection Scale used as an indicator of mother–infant connection/responsiveness. | 92 days (median 75, IQR 113) | EEG | 92 days (median 75, IQR 113) | Mother-infant connection was associated with increased frontal asymmetry. With more multisensory input, frontal asymmetry also shifted from negative (right asymmetry) to positive (left asymmetry). |
| Wade et al. (2024) | Romania | LO | 189 | Observational Record of the Caregiving Environment | 20.40 (7.20) months | EEG | Baseline: 20.40 (7.20) months, first follow-up: 30.84 (2.04) months, second follow-up: 42.36 (1.44) months | Caregiving quality significantly predicted lower averaged theta (but not alpha or beta) power (across waves), which in turn was negatively associated with later executive functioning (at 8 years of age). Caregiving quality was also associated with higher alpha power at baseline and lower theta power at baseline, and was associated with less growth (i.e., a less steep slope) in alpha over time. |
| Li et al. (2023) | US | CS | 35 (13 female) | SFP coded for facial expressions, vocalizations, and gaze direction | 2.73–3.9 months, 3.30 (0.33) | MRI, rs-fMRI | 2.73–3.9 months, 3.30 (0.33) | Bivariate correlations showed greater within-DMN FC at 3 months was associated with greater dyadic flexibility at 6 months. Similarly, greater within-SN FC at 3 months was associated with greater dyadic flexibility at 6 months. Lastly, greater DMN-SN anticorrelation (more negative values) at 3 months was associated with greater dyadic flexibility at 6 months; but not at 3 months. |
| Charpak et al. (2017) | Colombia | LO | 264 (148 female) | Kangaroo Mother Care (KMC): Continuous skin-to-skin contact between mother and infant, exclusive breastfeeding when possible; and early discharge and follow-up. | Infancy | MRI | 20 years | KMC participants who weighed ≤1800 g at birth had significantly larger cerebral volumes of total gray matter, cerebral cortex, and left caudate nucleus than control participants. |
| Hu et al. (2023) | US | CS | 35 (13 female) | SFP coded for facial expressions, vocalizations, and gaze direction, summed to reflect infant social engagement during the play and reunion episode | 2-5 months, 3.35 (0.41) | rs-fMRI | 2-5 months, 3.35 (0.41) | Greater positive within-network amygdala connectivity and greater positive amygdala-salience network connectivity predicted less infant social engagement during the reunion (did not survive correction for multiple comparisons). |

**Table 5**

Study characteristics of included studies measuring various other relational health measures

| **Study** | **Country** | **Design** | **N** | **Predictor measure** | **Predictor period** | **Imaging modality** | **Outcome period** | **Key Findings** |
| --- | --- | --- | --- | --- | --- | --- | --- | --- |
| Licata et al. (2015) | Germany | LO | 28 (15 female) | EAS | T1: 6.53–7.33 months, 6.95 (0.22)  T3: 49.73–52.80 months, 50.55 (0.67) | EEG | T2: 13.60–14.43 months, 13.98 (0.21) | Child EEG right frontal asymmetry score was negatively correlated with child involvement at 50 months, but not to the other EA-variables at 7 and 50 months of age. |
| Carver & Vaccaro (2007) | US | CS | 34 (14 female) | 6-minute interaction where new toys are introduced and infant is exposed to positive/negative/neutral emotional signal from parent (or experimenter), and references it | 12.0 months (0.25) | EEG | 12.0 months (0.25) | Infants showed an increase in ERP activity in response to stimuli associated with negative adult emotion. The amplitude of the P500 component at O2 in response to the negative stimulus was related to the change in infants’ interaction with their caregiver between the negative and neutral conditions, as well as a correlation with Nc latency to negative stimulus. |
| Mize & Jones (2012) | US | CS | 30 (17 female) | Mother alternating between playing with doll and ignoring child and play with child. Child's behavioural and affected responses were coded | 11–14.5 months, 12.90 (1.05) | EEG | 11–14.5 months, 12.90 (1.05) | A relationship emerged for approach-style responses in the rival-social condition and greater relative left frontal tonic EEG activity. Furthermore, a significant relationship was found between the jealousy profile (high versus low) and tonic left frontal EEG activity. Analyses for the central, parietal, and occipital regions EEG asymmetry values were not significant. |
| Mize et al. (2014) | US | CS | 35 (18 female) | Mother played with doll and ignored child. Child's behavioural responses were coded using mother directed gaze, gaze directed away, reach toward or away from mother | 6.75–10.75 months, 8.92 (0.99) | EEG | 6.75–10.75 months, 8.92 (0.99) | Comparing the book and doll condition revealed a nonsignificant trend toward more left mid-frontal asymmetry, a significant effect for more left lateral frontal asymmetry, as well as a significant effect for more right occipital asymmetry. Infants demonstrated greater relative left frontal EEG asymmetry during the jealousy-provoking doll condition than during the book condition and greater relative right occipital region activity. |
| Phillips et al. (2023) | UK | CS | 37 (18 female) | Coding of caregiver/infant gaze during three primary attention episodes - adult led looks to mutual attention, infant led looks to mutual attention, infant led looks to nonmutual attention | 11.12 months (1.33) | EEG | 11.12 months (1.33) | When caregivers joined their attentional focus, infants showed increased alpha suppression. No significant clusters were found when comparing neural oscillatory activity in the time before infant led and adult led mutual attention episodes. |
| Dawson et al. (1999) | US | CS | 117 (52 female) | Free play session with mother/infant coded for variables | 3.74 months (0.5) | EEG | 3.74 months (0.5) | For infants of depressed mothers, infants who exhibited reduced left frontal activation were less likely to be affectionate toward mother during free play and were more likely to grab mother’s clipboard or pen when she was filling out the questionnaire. For infants of nondepressed mothers, reduced left frontal activation was associated with higher levels of touching mother while she was filling out the questionnaire. No significant correlations between parietal EEG asymmetry and infant behaviour were found. |
| Marriott Haresign et al. (2023) | UK | CS | 55 (23 female) | Gaze onset type for mutual or non-mutual senders and receivers in 5-min free-play (mother and infant). | 12.2 months (1.47) | EEG, ERP | 12.2 months (1.47) | Significant ERP was found only for sender gaze onsets (when a person initiated gaze toward their partner) not for receivers (when a person receives gaze from partner). Inter-brain synchrony was not greater during mutual versus non-mutual gaze, |
| Minagawa-Kawai et al. (2009) | Japan | CS | 15 (7 female) | Infant shown stimuli of the mothers and unfamiliar-other's face in smiling or neutral expression | 9–13 months, 11.7 | fNIRS | 9–13 months, 11.7 | A significant difference in oxygenated haemoglobin changes between the smile and neutral stimuli was noted only in a medial prefrontal channel for the own-mother condition. A comparison of smile versus neutral between own and unfamiliar conditions yielded a significant difference. Analysis of deoxygenated haemoglobin did not show any statistical differences in any condition. |
| Smith et al. (2021) | UK | CS | 62 (21 female) | Mother-infant 5 minute unconstrained free-play session | 5.97 months (8.71 days) | EEG | 5.97 months (8.71 days) | There is a broad increase in oscillatory activity both when an infant played independently or interacted with their maternal figure comparative to when the infant was not exposed to any form of interaction. |
| Dégeilh et al. (2018) | Canada | LO | 28 (17 female) | Mother-infant 10-minute free-play (coded using Meins et al.’s (2001) rating for mind-mindedness); 15 months mother-infant puzzle sequence (coded using Whipple et al.’s (2011) rating for autonomy support) | T1: 13.09 months (1.39)  T2: 15.67 months (1.03) | rs-fMRI | 10.57 years (0.46) | Higher mind-mindedness was associated with significantly stronger negative connectivity (anti-correlation) between the right AI (SN) and two seeds of the DMN: vmPFC and the right angular gyrus. Higher autonomy support was associated with significantly stronger negative connectivity (anti-correlation) between the vmPFC (DMN seed) and the entire SN. |
| Safyer et al. (2020) | US | CS | 16 (7 female) | Maternal report of Parenting Stress Index-Short Form – 2nd subscale of Parental-child Dysfunctional Interaction | 215.7 days (31.5) | fNIRS | 215.7 days (31.5) | Infant negative emotionality and parent-child dysfunctional interaction were both found to be unique predictors of HbR activation during the happy faces condition. However, the interaction between these two variables was not significant. |
| Bagdasarov et al. (2025) | US | CS | 43 (20 female) | Infant- and mother-led joint eye gaze/attention during 4-min of free play | 5-11 months, 8.31 (1.53) | EEG | 5-11 months, 8.31 (1.53) | An increased rate of infant-led joint attention was associated with an increased duration of microstate 4 (fronto- central spatial topography). |
| Williams et al. (2025) | US | CS | 61 (33 female) | Parent-led joint eye-gaze/attention in 5-min free play | 248.56 days (13.22) | EEG | 248.56 days (13.22) | Significant positive association between parent alternating gaze and neural entropy in front/central regions. |
| Zhou et al. (2024) | China | CS | 27 (15 female) | Infant-directed speech (IDS; baby talk) in pseudoword learning task. | 16.48 months (1.60) | fNIRS | 16.48 months (1.60) | The L-dlPFC showed greater IDS than adult-directed speech responses whereas the IFG on both hemispheres showed opposite patterns, that is, significantly greater ADS than IDS responses. |

Note:

CS = Cross-sectional

LO = Longitudinal

RE = Retrospective

SSP = Strange Situation Procedure,
SFP = Still Face Paradigm,
HOME = Home Observation for the Measurement of the Environment,
EAS = Emotional Availability Scale (Biringen, 2008),
MACI = Manchester Assessment of Caregiver-Infant Interaction (Wan et al. 2012, 2013),
EQS = Attachment Behavior Q-Sort,
MRO = Mutually Responsive Orientation Scales
CNES = Coping with Children’s Negative Emotions Scale (Fabes et al., 2002)
CIB = Coding Interactive Behavior
CITMI–R = Early Mother-Child Interaction Coding System
AMBIANCE = Atypical Maternal Behavior Instrument for Assessment and Classification (Lyons-Ruth et al., 1999)

DMN = default mode network

SN = salience network

CEN = Frontal-parietal central executive network

Predictor/outcome assessment period is followed, where possible, according to Range, M, SD at the time of measurement.

**References**

Chajes, J. R., Stern, J. A., Kelsey, C. M., & Grossmann, T. (2022). Examining the Role of Socioeconomic Status and Maternal Sensitivity in Predicting Functional Brain Network Connectivity in 5-Month-Old Infants. *Frontiers in Neuroscience*, *16*, 892482. <https://doi.org/10.3389/fnins.2022.892482>

Gartstein, M. A. (2020). Frontal electroencephalogram (EEG) asymmetry reactivity: Exploring changes from baseline to still face procedure response. *International Journal of Behavioral Development*, *44*(3), 193–204. <https://doi.org/10.1177/0165025419850899>

Gartstein, M. A., Warwick, H., & Campagna, A. X. (2021). Electroencephalogram frontal asymmetry changes during emotion‐eliciting tasks and parent–child interaction dynamics. *Social Development*, *30*(2), 496–514. <https://doi.org/10.1111/sode.12484>

Hu, Y., Chen, H., Li, X., Larsen, R. J., Sutton, B. P., Gao, W., & McElwain, N. L. (2024). Associations between infant amygdala functional connectivity and social engagement following a stressor: A preliminary investigation. *Developmental Science*, *27*(1), e13418. <https://doi.org/10.1111/desc.13418>

Licata, M., Paulus, M., Kühn-Popp, N., Meinhardt, J., & Sodian, B. (2015). Infant frontal asymmetry predicts child emotional availability. *International Journal of Behavioral Development*, *39*(6), 492–496. <https://doi.org/10.1177/0165025415576816>

Mateus, V., Osório, A., Miguel, H. O., Cruz, S., & Sampaio, A. (2021). Maternal sensitivity and infant neural response to touch: An fNIRS study. *Social Cognitive and Affective Neuroscience*, *16*(12), 1256–1263. <https://doi.org/10.1093/scan/nsab069>

Minagawa-Kawai, Y., Matsuoka, S., Dan, I., Naoi, N., Nakamura, K., & Kojima, S. (2009). Prefrontal Activation Associated with Social Attachment: Facial-Emotion Recognition in Mothers and Infants. *Cerebral Cortex*, *19*(2), 284–292. <https://doi.org/10.1093/cercor/bhn081>

Mize, K. D., & Jones, N. A. (2012). Infant physiological and behavioral responses to loss of maternal attention to a social-rival. *International Journal of Psychophysiology*, *83*(1), 16–23. <https://doi.org/10.1016/j.ijpsycho.2011.09.018>

Mize, K. D., Pineda, M., Blau, A. K., Marsh, K., & Jones, N. A. (2014). Infant Physiological and Behavioral Responses to a Jealousy Provoking Condition. *Infancy*, *19*(3), 338–348. <https://doi.org/10.1111/infa.12046>

Pratik, M., & Robert C, M. (2006). Diffusion tensor imaging and tractography of human brain development. *Neuroimaging Clinics of North America*, *16*(1). <https://doi.org/10.1016/j.nic.2005.11.004>

Rahkonen, P., Heinonen, K., Pesonen, A., Lano, A., Autti, T., Puosi, R., Huhtala, E., Andersson, S., Metsäranta, M., & Räikkönen, K. (2014). Mother‐child interaction is associated with neurocognitive outcome in extremely low gestational age children. *Scandinavian Journal of Psychology*, *55*(4), 311–318. <https://doi.org/10.1111/sjop.12133>

Safyer, P., Volling, B. L., Wagley, N., Hu, X., Swain, J. E., Arredondo, M. M., & Kovelman, I. (2020). More than meets the eye: The neural development of emotion face processing during infancy. *Infant Behavior and Development*, *59*, 101430. <https://doi.org/10.1016/j.infbeh.2020.101430>

Smith, E. S., Elliott, D., Killick, R., Crawford, T. J., Kidby, S., & Reid, V. M. (2021). Infants Oscillatory Frequencies change during Free-Play. *Infant Behavior and Development*, *64*, 101612. <https://doi.org/10.1016/j.infbeh.2021.101612>

Stern, J. A., Kelsey, C. M., Yancey, H., & Grossmann, T. (2024). Love on the developing brain: Maternal sensitivity and infants’ neural responses to emotion in the dorsolateral prefrontal cortex. *Developmental Science*, *27*(6), e13497. <https://doi.org/10.1111/desc.13497>

Swingler, M. M., Perry, N. B., Calkins, S. D., & Bell, M. A. (2014). Maternal sensitivity and infant response to frustration: The moderating role of EEG asymmetry. *Infant Behavior and Development*, *37*(4), 523–535. <https://doi.org/10.1016/j.infbeh.2014.06.010>

Zhou, X., Wang, L., Hong, X., & Wong, P. C. M. (2024). Infant-directed speech facilitates word learning through attentional mechanisms: An fNIRS study of toddlers. *Developmental Science*, *27*(1), e13424. <https://doi.org/10.1111/desc.13424>
